# Optimal Control of Air Conditioning Systems by Means of CO_2_ Sensors in Electric Vehicles †

**DOI:** 10.3390/s22031190

**Published:** 2022-02-04

**Authors:** Luca Muratori, Lorenzo Peretto, Beatrice Pulvirenti, Raffaella Di Sante, Giovanni Bottiglieri, Federico Coiro

**Affiliations:** 1 Department of Electrical, Electronical and Information Engineering “Guglielmo Marconi”, Alma Mater Studiorum University of Bologna, 40136 Bologna, Italy; lorenzo.peretto@unibo.it; 2 Department of Industrial Engineering, Alma Mater Studiorum University of Bologna, 40136 Bologna, Italy; beatrice.pulvirenti@unibo.it (B.P.); raffaella.disante@unibo.it (R.D.S.); 3 Department of Engineering, Webasto Thermo & Comfort Italy S.r.l., 40062 Molinella, Italy; giovanni.bottiglieri@webasto.com (G.B.); federico.coiro@webasto.com (F.C.)

**Keywords:** CO_2_ sensors, Heating Ventilation and Air Conditioning (HVAC), energy saving, climate control load, Monte Carlo method (MCM)

## Abstract

Considering the consistent reduction in battery range due to the operation of the Heating Ventilation and Air Conditioning (HVAC) system, this study deals with the CO_2_ measurement inside the cabin of an electric crane and aims to reduce the energy consumption through the control of the air recirculation. A control strategy was defined and tested through an experimental set-up where the presence of a driver was simulated as a source of CO_2_. The cabin was placed inside a climatic wind tunnel and the benefits of this control strategy on the HVAC system energy consumption were assessed, both in the heating and the cooling modes. In addition, we discussed the optimal position of the CO_2_ sensor inside the cabin by comparing the results obtained from some sensors placed around the cabin occupant with the ones logged by three sensors in the breathing zone. Finally, an investigation of the uncertainty of the indirect measurement of the leakage flow and its dependence on the number of CO_2_ sensors installed in the cabin was made through the Monte Carlo method.

## 1. Introduction

In recent years, many researchers have focused their studies on the development of electric vehicles (EVs), due to the rising concerns about global warming and urban air pollution. It is known that internal combustion engine vehicles (ICEVs) are the main source of air pollutants in urban areas. As a result, in many countries the urban transport policies are encouraging the use of environmentally friendly vehicles. However, the driving range of EVs is limited in comparison to ICEVs, and the time needed to recharge the batteries is much longer than that for refilling the fuel tank. Moreover, the driving range is considerably reduced by the operation of the heating, ventilation, and air conditioning (HVAC) system. In particular, the comparison with ICEVs is relevant when the cabin compartment must be heated. While an internal combustion engine produces a lot of heat that can be recovered to heat up the cabin, the heat power dissipated by an electric engine is not enough. Therefore, the power needed by the HVAC system must be taken from the battery. As demonstrated by Lee et al. [1], in general the air conditioning (i.e., cooling and heating) is responsible for about a 33% average decrease in driving range for EVs.

A possible solution to reduce the energy consumption is to recirculate the cabin air. In fact, when the air that enters the HVAC unit is taken from inside the cabin, the air temperature differential is lowered in comparison to when it is drawn from outside the vehicle. Li et al. [2] tested a 2012 Prius PHEV (plug-in hybrid electric vehicle) with the Supplemental Federal Test Procedure SC03 driving cycle and, at the same time, measured the power absorbed by the HVAC system. Their results revealed that the air conditioner consumed 28% of the total power when the cabin air was recirculated, which was 6.1% less than in the fresh air mode. Another advantage of the air recirculation is that it isolates the driver and the passengers from the outside air and so reduces their exposure to the particulate matter. The decrease in the in-cabin particle concentration is obtained thanks to the fact that the air passes through the filter multiple times. In the test performed by Li et al. [2], it was found that the particle concentration was reduced by 85% when the air was recirculated.

However, during the air recirculation the CO_2_ concentration levels inside the cabin compartment tend to rise. This happens because the driver and the passengers are sources of CO_2_, and when the recirculation mode is on, the air exchanged with the outside ambient conditions is not enough to balance this generation. While the fresh air that comes from the external ambient conditions has a CO_2_ concentration of about 400 ppm, the air exhaled by a person generally contains from 38,000 ppm to 56,000 ppm [3] of CO_2_. Gladyszewska-Fiedoruk [4], fitting the CO_2_ mass balance model with the CO_2_ measurements performed inside a passenger car cabin, found that a driver and a passenger exhale 66 g/h and 35 g/h of CO_2_, respectively. Thus, the first exhales as much CO_2_ as a man during physical exercise, while the second that of a person during light sedentary work. Some researchers performed CO_2_ measurements in vehicle cabins and found the typical values reached in different kinds of vehicle cabins. According to Chiu et al. [5], the cabins of tour buses achieve maximum CO_2_ concentrations of more than 3000 ppm and maximum daily average concentrations of 2510.6 ppm for the driver zone and 2646.9 ppm for the passenger zone. Regarding passenger vehicles, Lee and Zhu [6] measured in-cabin CO_2_ concentrations that ranged from 620 ppm to 930 ppm in the fresh air mode, while they reached levels of 2500–4000 ppm in the recirculation mode. Zhu et al. [7] showed how quickly the CO_2_ concentration levels can grow in a confined space such as a vehicle cabin. In particular, they observed that the CO_2_ concentration reached levels of 4500 ppm in less than 10 min when the cabin air was recirculated.

The CO_2_ concentration levels generally encountered in vehicle cabins are not high enough to be considered harmful to humans. However, according to the study of Satish et al. [8], the exposure of human subjects to 1000 ppm and 2500 ppm for 2.5 h causes relatively moderate and large decrements in decision-making performance. Therefore, in the case of a cabin vehicle of any type, maintaining the indoor CO_2_ concentration levels under certain limits is important to prevent accidents due to the lack of attention of the driver. According to ANSI/ASHRAE Standard 62.1 [9], the CO_2_ concentration in indoor environments should not exceed 700 ppm above outdoor air levels. Considering that the outdoor CO_2_ concentration is ~400 ppm, the limit for indoor environments should be 1100 ppm. NIOSH [10] recommended an exposure limit equal to 5000 ppm of CO_2_ for a 10 h workday during a 40 h workweek and a short-term exposure limit of 30,000 ppm.

In the present study, to reduce the HVAC energy consumption, while maintaining the CO_2_ levels within safety conditions, we implemented an automatic on/off control strategy of the air recirculation in the cabin of an electric crane. Then, we monitored experimentally the energy saved thanks to this control strategy, in comparison with the operation in a fresh air mode, during tests performed with different ambient conditions in a climatic wind tunnel [11]. The cabin of an electric crane was chosen for our tests due to its small dimensions, suitable for fitting in a small, available climatic wind tunnel, which also made it possible to keep the costs of the experiment low. Moreover, such a solution represents one of the vehicles for which Webasto designs their HVAC systems. In a recent article, Pan et al. [12] made an annual energy consumption model that calculated the outside ventilation rate in an electric vehicle, based on a CO_2_ concentration limit of 1100 ppm and the windshield anti-fog requirements. Consequently, they evaluated the energy saving effect of this strategy in 30 cities across China. Their results showed that utilizing the recirculated air, while maintaining the CO_2_ levels within the requirements, can extend the driving range by 11–30% through a year.

Pei et al. [13] demonstrated, both through CFD (Computational Fluid Dynamics) simulations and experiments, that the CO_2_ concentration is not evenly distributed across a mechanically ventilated room. Furthermore, they proved that its diffusion changes depending on the ventilation settings. Considering that the required CO_2_ concentration levels should be maintained in the air that the occupant is inhaling, in this study we defined a parameter ([14]) to assess the optimal location for the CO_2_ sensor inside the cabin.

Finally, we used the Monte Carlo method ([15]) to compute the uncertainty in the leakage flow measurement depending on the number of CO_2_ sensors installed in the cabin compartment. The leakage flow represents the exchanged air flow between the cabin compartment and the external ambient conditions. It can be calculated from the CO_2_ measurements, inside and outside the cabin, through the CO_2_ mass balance equation (Jung, 2013 [16]; Jung et al. 2017 [17]). The purpose of this last work was to give an indication of the maximum number of sensors that, in our opinion, it is convenient to install in the cabin.

## 2. Materials and Methods

For the present study, the CO_2_ concentration measurements were performed in a ~2 m^3^ cabin of an electric crane, which was placed inside a climatic wind tunnel. In this environment, different ambient conditions could be established by choosing a combination of the set points for ambient temperature, relative humidity, solar radiation, and wind speed. The ranges considered for these parameters were 10 °C to 30 °C for the ambient temperature, 50% to 60% for the relative humidity, and 590 W/m^2^ to 685 W/m^2^ for the solar radiation, and the wind speed was fixed at 15 km/h. The cabin HVAC system was made of an electric heater, a refrigeration cycle, a fan, and two flaps. One flap was used to decide whether to induct fresh air or recirculated air in the cabin, while by changing the position of the other, we could vary how the air was distributed to the different vents in the cabin. The electric heater installed inside the HVAC system was a positive temperature coefficient (PTC) heater. Then, when it heats up, the resistivity of the material increases, causing a reduction in the current absorbed and therefore of the heating power produced. The PTC heater was made of three heating elements; so, it could be regulated on three levels of heating power when it was on: 0.8 kW, 1.6 kW, and 2.4 kW. The heating power was controlled using a PI controller on the cabin temperature error: the difference between the set-point temperature and the actual cabin temperature. Another PI controller on the cabin temperature error was used to control the rotational speed of the compressor for the refrigeration cycle. While the PTC heater could be turned off when the actual temperature was inside a set-point temperature interval, the compressor could not: a minimum speed of 800 rpm was set for the purpose of cabin dehumidification. Moreover, a limit on the maximum compressor speed was set to prevent icing on the evaporator (the temperature of the evaporator had to be greater than 4 °C). The cabin manufacturer placed the temperature sensor that delivers the data of the cabin temperature to the HVAC control unit in the front right space of the cabin, at a height of 0.45 m with respect to the floor level. The resolution of the sensor embedded in the cabin was 0.5 °C. We then added a type-j thermocouple, with the sensor in the same position as the first one. The temperature measurements reported in the present paper are referred to the type-j thermocouple. This sensor could measure temperatures in the range of 0−760 °C with an accuracy of 2.2 °C or 0.75% of the reading, whichever was bigger.

During the experiment, in place of the driver, a manikin was seated in the cabin. To simulate the breathing of the person, in particular the carbon dioxide generated in the cabin during his exhalation, a system to inject CO_2_ in the cabin was built. We used a pressurized tank as the source of CO_2_, and the gas flow from the tank to the cabin was controlled thanks to these components put in series: a pressure regulator, a needle valve, and a thermal mass flow sensor. The CO_2_ was injected into the cabin near the mouth of the manikin, and the flow was fixed at the value of 66 g/h. Gladyszewska-Fiedoruk [4] stated through experimental CO_2_ measurements in the cabin of a passenger car, that a passenger is responsible for a CO_2_ generation equal to a person who is doing light sedentary work (35 g/h), while a driver exhales as much CO_2_ as during physical exercise (66 g/h). In this study, the thermal mass flow sensor used to measure the CO_2_ mass flow injected into the cabin was the Sensirion SFM 4100 [18]. This is a low-cost digital mass flow meter for gasses which is able to measure up to 20 slm (reference condition for standard liter per minute: 20 °C, 1013 mbar) with an accuracy of the 3% of the reading or 0.15% of the full scale, whichever is bigger. The sensor element, the signal processing, and the digital calibration are combined on a single microchip embedded in the gas flow meter. In addition to the thermal mass flow sensor, this chip contains an amplifier, an A/D converter, an EEPROM memory, and a digital signal processing circuitry and interface. Through its digital I2C interface, the gas flow meter was embedded into a microprocessor environment (Arduino 2560 Mega).

For the measurement of the CO_2_ concentration, the non-dispersive infrared (NDIR) sensors Telaire^®^ T6713-5k and Telaire^®^ T6743-40K-E [19], produced by Amphenol Corporation in Pennsylvania, U.S., were used. NDIR sensors work by doing a specific spectral analysis within the infra-red wavelengths; carbon dioxide absorbs light at a 4.2 μm wavelength. The components of these sensors are an incandescent lamp, used as the infra-red source, a light-detecting thermopile with a narrow bandpass filter, and a gas chamber, located between the source and the detector of the IR radiation. In essence, the more CO_2_ there is in the chamber, the less light is received by the thermopile. By filtering the light over the area around the wavelength equal to 4.2 μm, it is possible to observe a change in the output signal as there is more or less CO_2_. The correlation between the CO_2_ concentration and the difference between the intensity of the radiation emitted by the IR source and of the light detected by the thermopile is given by the Beer’s Law [20]. The Telaire^®^ T6713-5k is a sensor which is ideal for application in an indoor environment where the CO_2_ levels need to be measured and controlled for air quality and energy-saving requests. For this purpose, the sensor is factory-calibrated to measure CO_2_ concentration levels up to 5000 ppm. In the measurement range, the accuracy is ±30 ppm ± 3% of the reading. To acquire the output signal, we used the digital I2C interface so that the CO_2_ sensor could be embedded into a microprocessor environment. The Telaire^®^ T6743-40K-E is a CO_2_ sensor used for automotive HVAC applications. In particular, it has two purposes, one for safety and the other for energy saving. For the first purpose, the sensor is used to measure and control the in-cabin CO_2_ levels to prevent driver drowsiness; in this case, the sensor can be configured to measure CO_2_ concentrations up to 40,000 ppm with an accuracy of ±200 ppm ± 10% of the reading. The second purpose is achieved through demand-control ventilation, based on a target in-cabin CO_2_ concentration level. In this situation, the sensor can be configured to operate in the range 400–5000 ppm. In order to communicate with the control unit of a vehicle HVAC system, the T6743-40K-E model implements an LIN interface. Both the models of CO_2_ sensors used in this study have received a factory calibration and use an algorithm called ABCLogic (Automatic Background Calibration) to compensate sensor long-term drift. Outside levels of CO_2_ are generally around 400 ppm. When an indoor environment is unoccupied for 4 to 8 h, the CO_2_ levels will tend to drop to outside background levels. ABCLogic utilizes the computing power in the sensor’s on-board microprocessor to remember the lowest CO_2_ concentration that takes place every 24 h. The sensor assumes this low point is at outside levels. Once the sensor has collected 14 days’ worth of low concentration points, it performs a statistical analysis to see if there have been any small changes in the sensor reading over the background level that could be attributable to sensor drift. If the analysis concludes there is drift, a small correction factor is made to the sensor calibration to adjust for this change.

**Figure 1:**
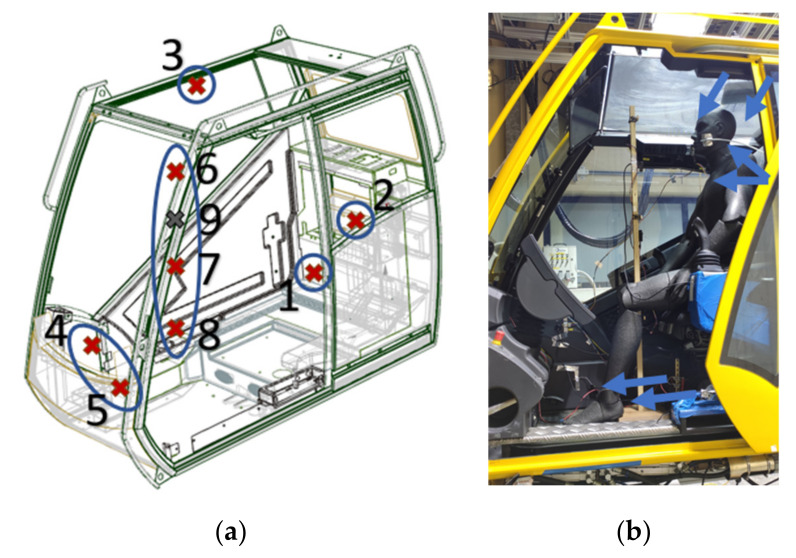
View of the crane cabin: (**a**) schematic representation of the sensors positions; (**b**) view of the vent positions.

The CO_2_ concentration in the cabin volume was measured with 9 sensors placed in 9 different locations, as represented schematically in Figure 1a.

Considering the values of height with respect to the level of the floor where the occupant lays the feet, the sensor locations were the following:

1. At the rear bottom space of the cabin, in the proximity of the recirculation vent, at a height of 0.36 m.
2. In the middle-left space of the cabin, at a height of 0.96 m.
3. Near the roof, in the top right space of the cabin, at a height of 1.41 m.
4. In the front-bottom space of the cabin, on the wall at the right of the center console, at a height of 0.45 m.
5. In the same area as the previous sensor, but at a height of 0.30 m.
6. The last 4 sensors were in the middle of the volume in front of the manikin, all on the same vertical axis, at the heights of 0.45 m, 0.92 m, 1.16 m, and 1.40 m. We thought that in these positions we would have been able to measure the CO_2_ concentration level of the air inhaled by a possible driver. In fact, as described by ASHRAE 62.1 [9], the volume included by the planes at 0.075 m and 1.8 m above the floor, and a surface obtained from a 0.6 m offset of the lateral walls or of fixed air conditioning equipment is defined as the “breathing zone”. However, the last condition could not be satisfied because the horizontal dimensions of the cabin were too small. Even putting the sensors in the middle of the cabin, the distance between them and the walls on both sides was 0.45 m.

A good position for the sensor could be in the cabin seating. However, this position has not been considered in this paper because it is not easy to implement in a real application. In particular, (i) the CO_2_ reading could be perturbed by the occupant’s emission; (ii) the sensor reading could be perturbed by the seat temperature in the case of a heated seat; and (iii) a custom-made seat would be necessary in order to place the sensor and the cable inside it. Of all the CO_2_ sensors installed in the cabin, only the one identified with the number 9 in Figure 1a was of the type Telaire^®^ T6743-40K-E, while the other 8 sensors were of the model Telaire^®^ T6713-5k. The sensor number 9, through its LIN interface, could communicate the value of the CO_2_ concentration measured to the HVAC control unit. Thanks to this information, the demanded ventilation of fresh air was controlled by varying the angular position of the recirculation flap. The control strategy that we implemented provided only two positions for the recirculation flap. In the first one, the flap, whose angle of inclination was 0° with respect to the horizontal direction, completely closed the passage for the recirculated air. Thus, the HVAC system would have processed only fresh air. The opposite would have happened with the flap in the second position, with its angle of inclination equal to 90° with respect to the horizontal direction. In this case the HVAC system would have processed only the recirculated air. For the remnant sensors, defined in Figure 1a with the numbers 1 to 8, the model Telaire^®^ T6713-5k was chosen for its higher accuracy with respect to the other model and for its digital I2C interface. As the aim of these sensors was only to log the data of the measurements, we did not have the need for an LIN interface that would have allowed them to communicate with the HVAC control unit. Moreover, with the digital I2C interface the output signal from the sensors could be easily acquired using a microprocessor. In our case, an STM32F411RE microcontroller was utilized, and we mounted the 8 NDIR CO_2_ sensors on two I2C buses; so, there were 4 slave devices on each bus.

### 2.1. Automatic Air Recirculation Control Strategy

In the present study, a control strategy for the position of the recirculation flap was implemented. In this way, we could control the amount of air exchanged between the cabin’s internal volume and the external ambient conditions (here it was the volume of the climatic wind tunnel) in order to contain the in-cabin CO_2_ concentration levels within a certain interval of values. In this experiment, the input of the control strategy was the CO_2_ measurement inside the cabin, whose reading was from the sensor labelled with the number 9 in Figure 1a. Concerning this aspect, ASHRAE 62.1 [9] states that the upper limit for the CO_2_ concentration level in an indoor environment is 700 ppm over the outdoor CO_2_ concentration level, which is generically equal to about 400 ppm. In this study, the control strategy that was implemented in the HVAC control unit did not allow a mixture of fresh air and recirculated air. However, the recirculation flap position could only be switched from the recirculation mode to the fresh air mode and vice versa. At the start of the HVAC system, the control unit would have kept the recirculation flap in the position of recirculation mode; then, if the Telaire^®^ T6743-40K-E sensor had detected a CO_2_ concentration level higher than 1100 ppm, the system would have been switched to the fresh air mode. This configuration would have been maintained until the measured CO_2_ concentration level had dropped to under 900 ppm; at this point, the HVAC control unit would have restored the recirculation flap to the recirculation mode position. In essence, we built an on/off control strategy. The aim of the introduction of a CO_2_ sensor to a vehicle cabin is to help reduce the energy consumption of the HVAC unit, as the heating or cooling recirculated air generally requires much less energy because the temperature differential is much less. In this study, we carried out the tests listed in Table 1 to determine the energy saved due to the introduction of the controlled outside air induction, as described.

The measurements of the current absorbed by the HVAC system were made with two Hall-effect probes (Fluke i1010 AC/DC Current Clamps). One was used for the compressor, which worked under a voltage of 400 V, while the other was used for all the remnant elements (fan, electric heater, and cabin electronic components), which operated under a voltage of 27 V. Before the start of each experiment, the cabin door was kept open in order to have the initial conditions of the CO_2_ concentration and temperature in the cabin air similar to the external ambient conditions. In the HVAC control unit, two configurations of the flap for the distribution of the air to the different cabin vents were set. In the heating mode, all the air was blown through a diffuser positioned under the seat. While in cooling mode, the air flow was divided among the same diffusers, with the vents near the roof and the vents at the bottom of the lateral window, as shown in Figure 1b. In both cases the fan speed was fixed at 50% of its maximum; only in tests 1a and 2a was it fixed at 80%.

### 2.2. Assessment of the Optimal CO_2_ Sensor Position

To investigate the optimal location for the CO_2_ sensor, the measurements performed by the sensors placed in the breathing zone, defined with the numbers 6, 7, and 8 in Figure 1a, were used as the reference values. These results were compared, at each time instant, to the CO_2_ concentration levels recorded by the five sensors scattered around the cabin, identified in Figure 1a with the numbers 1 to 5. The purpose was the evaluation of which one of these five positions for the sensor was the best to monitor the in-cabin CO_2_ concentration levels. In particular, our aim was to assess which one of these five sensors could measure the closest CO_2_ concentration levels to the interval of values logged in the breathing zone, in all the working conditions analysed. Therefore, a parameter was defined to express the positioning performance by quantifying the offset between the measurements made in the spots 1 to 5 and the CO_2_ concentration interval measured in the breathing zone. We defined the extremes of this interval as:

(1) $$C_{max}\left(t\right)=max\left(C_{6}\left(t\right),\;C_{7}\left(t\right),\;C_{8}\left(t\right)\right)\;C_{min}\left(t\right)=min\left(C_{6}\left(t\right),\;C_{7}\left(t\right),\;C_{8}\left(t\right)\right)$$

where t is the time instant, $C_{i}$ is the CO_2_ concentration measured by the sensor i, $C_{max}$ and $C_{min}$ are, respectively, the maximum and minimum CO_2_ levels in the breathing zone. Considering the spots 1 to 5 (for i = 1, …, 5), we calculated, at each time instant, the offset ($\delta_{i}\left(t\right)$) of the measurement made by sensor i with respect to the breathing zone CO_2_ levels as:

(2) $$if\;C_{i}\left(t\right)<C_{min}\left(t\right)\to \delta_{i}\left(t\right)=C_{min}\left(t\right)-C_{i}\left(t\right)\;if\;C_{i}\left(t\right)>C_{max}\left(t\right)\to \delta_{i}\left(t\right)=C_{i}\left(t\right)-C_{max}\left(t\right)\;if\;C_{min}\left(t\right)<C_{i}\left(t\right)<C_{max}\left(t\right)\to \delta_{i}\left(t\right)=0$$

We divided $\delta_{i}\left(t\right)$ by the mean value of the CO_2_ concentrations measured at time t by sensors 6, 7, and 8 to calculate the offset in the percentage ($\Delta_{i}\left(t\right)$):

(3) $$\Delta_{i}\left(t\right)=100\frac{\delta_{i}\left(t\right)}{mean\left(C_{6}\left(t\right),C_{7}\left(t\right),\;C_{8}\left(t\right)\right)}$$

We calculated the mean value (${\bar{\Delta }}_{i}$) of $\Delta_{i}\left(t\right)$ over the time duration of each test (τ = 2400 s) to assess the sensor positioning performance:

(4) $${\bar{\Delta }}_{i}=\frac{1}{\tau }\int_{0}^{\tau }\Delta_{i}\left(t\right)\;dt$$

Finally, we assumed that the smaller the value of ${\bar{\Delta }}_{i}$, the better the performance of the spot i. The settings of the climatic wind tunnel and of the HVAC system for the tests that we performed are listed in Table 2. The working conditions examined were the heating, ventilation, and cooling modes, with the fan speed configured at 50% of its maximum. Moreover, for each of these modes, we made the HVAC system operate in the fresh air mode and with the control strategy for the recirculation flap that was described previously. Before the start of each experiment, the cabin door was kept opened in order to have the initial conditions of the CO_2_ concentration levels in the cabin air similar to the external ambient conditions.

### 2.3. Determination of the Measurement Uncertainty of the Leakage Flow

Some standards, related to vehicle air conditioning, define the minimum fresh air flow that must be introduced in the cabin compartment. For example, the standard VDV 236 [21], which refers to buses, says that the HVAC system has to be designed in such a way that at least 15 m^3^/h per person of fresh air must be provided. With the Monte Carlo method (MCM), we can evaluate accuracy with which the leakage flow can be estimated from the CO_2_ measurement into the cabin compartment. The measurement uncertainty of the leakage flow was used to consider the maximum number of CO_2_ sensors that is convenient to install inside the cabin compartment.

#### 2.3.1. Equations Used in the MCM

The MCM, which is defined in Supplement 1 to the Guide to the expression of Uncertainty in Measurements (GUM) [15], was used to determine the estimate and the uncertainty of the measurement of the leakage flow, which could be obtained indirectly from the measurements of the CO_2_ concentration levels and of the source of CO_2_ in the cabin. The leakage flow is the product between the air exchange rate (AER) and the cabin volume ($V_{cab}$); in essence, it is the fresh air flow that enters the cabin, which is also equal to the air flow exhausted by the cabin. The model that correlates the leakage flow (Q) to the mean CO_2_ concentration ($C_{cab}$) in the cabin volume is this mass balance equation:

(5) $$V_{cab}\frac{dC_{cab}}{dt}=C_{amb}Q-C_{cab}Q+S$$

where $C_{amb}$ is the CO_2_ concentration level in the external ambient conditions, S is the source of CO_2_, and t is the time instant. With the kind of sensors described in Section 2, the measurable quantities are $C_{cab}$, $C_{amb}$, and S. Thus, in this equation there are 2 unknowns; these are the leakage flow Q and the cabin volume $V_{cab}$. According to Jung [16], when a vehicle is motionless, and the wind speed is therefore zero, and the ventilation fan is off, the leakage flow is negligible and the mass balance Equation (5) for the CO_2_ becomes, after integrating in time:

(6) $$C_{cab}=\frac{S}{V_{cab}}t+C_{cab,0}$$

where $C_{cab,0}$ is the initial value of the CO_2_ concentration in the cabin. In this particular condition, we obtain a linear equation with only one unknown, which is the cabin volume. Therefore, from Equation (6), considering two time instants, $t_{1}$ and $t_{2}$, it is possible to find $V_{cab}$:

(7) $$V_{cab}=\frac{10^{6}Q_{CO_{2}}\;\left(t_{2}-t_{1}\right)}{C_{cab,2}-C_{cab,1}}$$

where $C_{cab,1}$ and $C_{cab,2}$ are the mean CO_2_ concentration levels in the cabin volume, respectively, at time $t_{1}$ and $t_{2}$. S has been substituted with $10^{6}Q_{CO_{2}}$, where $Q_{CO_{2}}$ is the CO_2_ mass flow injected into the cabin and measured with the mass flow meter. A test was performed where the CO_2_ concentration levels were measured with the cabin in this condition. With the results obtained from the measurements at time $t_{1}$ and $t_{2}$, we applied the MCM to find the mean and the standard deviation estimations of the cabin volume. We defined M to be equal to 3 × 10^6^, the number of Monte Carlo trials. Thus, we generated a sequence of M values for each input quantity ($Q_{CO_{2}}$, $C_{cab,1}$ and $C_{cab,2}$) by performing M random sampling from their probability distributions. Consecutively, we obtained a sequence of M values for $V_{cab}$ from the calculation of Equation (7) for the M trials. We did not corrupt the MCM with the quantities $t_{1}$ and $t_{2}$ because the error on the timestamp is negligible with respect to the other measurements. The variables $C_{cab,1}$ and $C_{cab,2}$ are obtained from the mean value of the measurements made by the sensors ($C_{i,1}$ and $C_{i,2}$, where i is the sensor counter) placed inside the cabin. The reason is that Equation (7) is a lumped-parameter model; so, only one value of the CO_2_ concentration level is considered for the entire cabin. For each input quantity, we considered a rectangular distribution, which means the error is uniformly distributed inside the interval of accuracy of each sensor, as given by the datasheet. The output signals of both the CO_2_ sensors and the mass flow meter were already processed by their on-board microchips; the microprocessors (Arduino 2560 Mega and STM32F411RE) received a message of zeros and ones from the slave devices. Therefore, no other errors were added to the ones specified by the datasheets of the sensors. The choice of considering the error as uniformly distributed is motivated by the fact that the manufacturer of the sensors did not provide a PDF (Probability Density Function) associated with the accuracy, but only an interval. As stated in the GUM [22], if the only available information regarding a quantity X is a lower limit a and un upper limit b with a<b, then, according to the principle of maximum entropy, a rectangular distribution $R\left(a,b\right)$ over the interval $\left[a,b\right]$ would be assigned to X. Let us define $x_{j}$ as a generic input quantity with $a_{j}$ and $b_{j}$ as its lower and upper limits, respectively. We can write the generic element ${\hat{x}}_{j}\left(m\right)$ of the corrupted sequence ${\hat{x}}_{j}$ as:

(8) $${\hat{x}}_{j}\left(m\right)=a_{j}+\left(b_{j}-a_{j}\right)r_{j}\left(m\right)$$

where m is the element counter and $r_{j}\left(m\right)$ is a random draw from the standard rectangular distribution whose lower and upper limits are 0 and 1, respectively. As $C_{cab,1}$ ($C_{cab,2}$) was calculated as the mean value of the measurements made in $N_{s}$ spots inside the cabin, the element of the corrupted sequence ${\hat{C}}_{cab,1}$ can be written as:

(9) $${\hat{C}}_{cab,1}\left(m\right)=\frac{1}{N_{s}}\overset{N_{s}}{\underset{i=1}{\sum }}a_{C_{i,1}}+\left(b_{C_{i,1}}-a_{C_{i,1}}\right)r_{C_{i,1}}\left(m\right)$$

where $a_{C_{i,1}}$ and $b_{C_{i,1}}$ are the lower and upper limits for $C_{i,1}$ (CO_2_ measurement in the spot number i). The same formula can also be written for ${\hat{C}}_{cab,2}\left(m\right)$. Substituting the input quantities in Equation (7) with the respective M corrupted values, we obtained a sequence of M corrupted elements ${\hat{V}}_{cab}\left(m\right)$. Finally, from these M samples of the cabin volume obtained through the MCM, we could calculate the estimation of the mean value and of the standard deviation for the cabin volume: $\mu_{V_{cab}}$ and $\sigma_{V_{cab}}$.

At this point, in Equation (5) only Q remains unknown. Integrating in time Equation (5) and rewriting it considering 2 time instants, $t_{1}$ and $t_{2}$, we have:

(10) $$C_{cab,2}=\left(C_{cab,1}-C_{amb}-\frac{10^{6}Q_{CO_{2}}}{Q}\right)\exp\left[\frac{Q}{V_{cab}}\left(t_{2}-t_{1}\right)\right]+C_{amb}+\frac{10^{6}Q_{CO_{2}}}{Q}\;$$

The same procedure used for the cabin volume was now repeated to find the estimate and the coverage interval for the leakage flow Q. This time, we performed a test with the ventilation fan speed fixed at 50% of its maximum, where the CO_2_ concentration levels were measured in the various spots inside the cabin and in the external ambient conditions; moreover, the CO_2_ mass flow injected into the cabin was measured. We used rectangular distributions for the input quantities $C_{i,1}$, $C_{i,2}$, $C_{amb}$, and $Q_{CO_{2}}$. As shown in Section 3.3, a Gaussian distribution was obtained for $V_{cab}$; so, the element of the corrupted sequence ${\hat{V}}_{cab}$ was calculated at each trial of the MCM as:

(11) $${\hat{V}}_{cab}\left(m\right)=\mu_{V_{cab}}+\sigma_{V_{cab}}z_{V_{cab}}\left(m\right)$$

where $z_{V_{cab}}\left(m\right)$ is a random draw from the standard Gaussian distribution that has the best estimate equal to 0 and the variance equal to 1. Finally, substituting in Equation (10) the input quantities with their corrupted sequences ${\hat{C}}_{cab,1}$, ${\hat{C}}_{cab,2}$, ${\hat{C}}_{amb}$, ${\hat{Q}}_{CO_{2}}$, and ${\hat{V}}_{cab}$, a sequence $\hat{Q}$ of M elements was obtained by solving Equation (10) numerically. From these M samples of the leakage flow, we could calculate the mean value ($\mu_{Q}$) and the 95% coverage interval for $\hat{Q}$.

The code used to apply the MCM to Equations (7) and (10), was written in the Matlab environment. Moreover, to calculate the mean value and standard deviation of a variable, the Matlab tools mean and std were used, respectively. Another Matlab tool, fzero, was utilized to find numerically the value of $\hat{Q}\left(m\right)$ that solves Equation (10) at each Monte Carlo trial. Finally, the probability distribution of each variable was obtained thanks to the Matlab tool histogram.

#### 2.3.2. Parameters of the Tests

For this examination, two types of tests were analysed. The first was to find the PDF for the cabin volume, while the second was to compute the PDF for the leakage flow. The settings for the cabin and the climatic wind tunnel are listed in Table 3.

Each one of these tests was carried out 8 times. From the first to the eighth case, we changed the number of CO_2_ sensors considered inside the cabin from 1 to 8 sensors, respectively. The aim was to observe how the uncertainty in the prediction of the fresh air flow that enters the cabin is affected by the number of CO_2_ sensors installed in the cabin. The errors relative to the measurements made in each test are listed in Table 4.

The time intervals between $t_{1}$ and $t_{2}$ in test #1c and test #2c were, respectively, 700 s and 2447 s.

## 3. Results

In this section, a description of the experimental results is provided for the three topics explained previously. In the first part, a comparison is made between the results of the energy consumed by the HVAC system when working in the fresh air mode and when operating with the automatic control of the fresh air inducted into the cabin activated. In the second part, we show how the CO_2_ concentration varies in the breathing zone and around the rest of the cabin volume. Also evaluated is the optimal position for the CO_2_ sensor among the five spots analysed, thanks to the introduction of a parameter. In the third part, the results of the application of the Monte Carlo method to find the estimate and the uncertainty of the two indirect measurements, the cabin volume and the leakage flow, are shown. 

### 3.1. Assessment of Energy Saving Due to the Automatic Air Recirculation Control Strategy

#### 3.1.1. Heating Mode

Test #1a and test #3a were executed in the fresh air mode, while during test #2a and test #4a, the cabin air recirculation was automatically controlled, as described in Section 2.1. The ambient temperature in the climatic wind tunnel was set at 10 °C for test #1a and test #2a, and at 15 °C for test #3a and test #4a.

**Figure 2:**
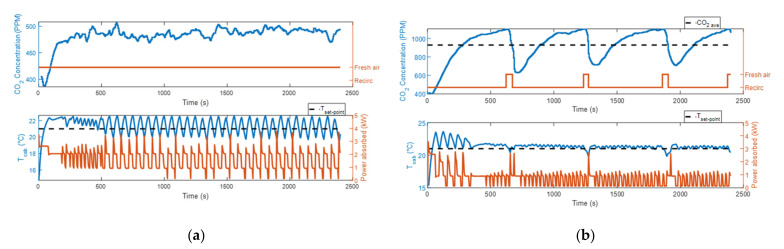
CO_2_ concentration, power absorbed by the HVAC system and cabin temperature measurements at ambient temperature equal to 10 °C: (**a**) test #1a; (**b**) test #2a.

**Figure 3:**
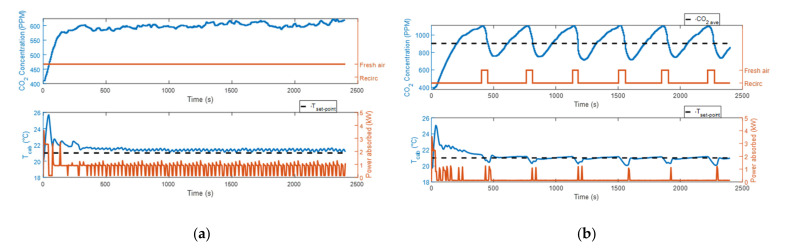
CO_2_ concentration, power absorbed by the HVAC system and cabin temperature measurements at ambient temperature equal to 15 °C: (**a**) test #3a; (**b**) test #4a.

Figure 2a,b and Figure 3a,b show that in the first two tests the CO_2_ concentration levels measured were lower compared to the other tests, due to the higher fan speed. In particular, it was equal to 80% of the maximum value in the first case and 50% in the second. As the maximum CO_2_ concentration level is limited at 1100 ppm when the air recirculation control strategy is activated, during test #4a the HVAC control unit triggered the fresh air mode more frequently than during test #2a. Looking at the temperature plots in Figure 2a,b and Figure 3a,b, when the system operated in the fresh air mode and the steady-state condition was reached, the cabin temperature kept oscillating with almost the same amplitude around a constant value. Conversely, when the air recirculation control strategy was activated, a small temperature drop was recorded every time the fresh air mode was turned on. In both test #2a and test #4a, the system could work for most of the time in recirculation mode, and consequently, we obtained a considerable reduction in the energy consumption with respect to test #1a and test #3a, respectively. When the ambient temperature was 10 °C, the energy consumed by the HVAC system was 1.092 kWh at the end of test #1a and 0.505 kWh at the end of test #2a. Therefore, the air recirculation control strategy allowed an energy saving of the 53.8%. When the ambient temperature was 15 °C, the energy consumed by the HVAC system was 0.514 kWh at the end of test #3a and 0.141 kWh at the end of test #4a. Therefore, the air recirculation control strategy allowed an energy saving of the 72.6%. The reason behind this high value of energy consumption reduction can be found by looking at the plot at the bottom of Figure 3b. The figure shows that, when the recirculation mode is on, the cabin temperature curve is not decreasing and at the same time the PTC heater is not delivering any heating power. In these instants, the only source of heat for the cabin is the solar radiation. Therefore, we can understand that the heat dissipated by the cabin, due to the 6 °C of temperature difference between the inside and the outside environments, is balanced by the solar radiation. As shown in Figure 3b, the control system kept the PTC heater off for almost all the duration of test #4a, and it turned the heater on only in those short instants when the fresh air mode was activated. Every time the fresh air mode is turned on, the HVAC system starts to introduce air at 15 °C into the cabin, and therefore, a fall of the cabin temperature is observed. When the HVAC control unit notices this change, it sends the command to activate one of the heater’s heating elements.

#### 3.1.2. Cooling Mode

**Figure 4:**
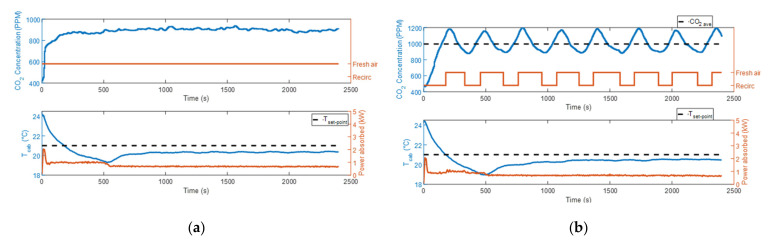
CO_2_ concentration, power absorbed by the HVAC system and cabin temperature measurements at ambient temperature equal to 23 °C: (**a**) test #5a; (**b**) test #6a.

**Figure 5:**
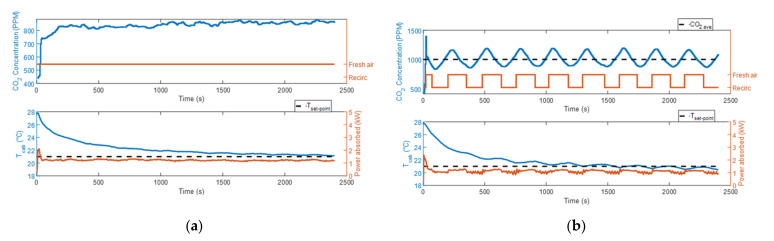
CO_2_ concentration, power absorbed by the HVAC system and cabin temperature measurements at ambient temperature equal to 30 °C: (**a**) test #7a; (**b**) test #8a.

With the ventilation distribution set in cooling mode, we measured higher CO_2_ concentration levels with respect to the heating mode cases, even if the fan speed was the same (50% of the maximum value), as shown in Figure 4 and Figure 5. The reason was probably an accumulation of CO_2_ in the breathing zone, where the CO_2_ sensor was placed (sensor 9 in Figure 1a), due to the different internal movement of air. When the air recirculation control strategy was used, this caused a faster increase in the measured CO_2_ levels when the air recirculation mode was on and a slower decrease when the fresh air mode was taking place. This phenomenon triggered a more frequent on/off of the recirculation mode, with respect to the heating condition, as shown in Figure 4b and Figure 5b. When the air recirculation control strategy was on, the HVAC system spent about half the time of the experiment in the fresh air mode. This implied that the energy saving could not be as great as in the heating mode.

In test #5a and test #6a, the ambient temperature (23 °C) was close to the cabin set-point temperature. In both cases, the steady-state conditions were reached with an actual cabin temperature lower than the set-point temperature. At the steady-state condition, the compressor worked constantly at the minimum rotational speed (800 rpm), delivering more power than needed. So, the cabin temperature measurements and the energy consumption obtained in these two experiments were very similar. The HVAC system consumed 0.502 kWh in test #5a and 0.498 kWh in test #6a. Therefore, only 0.8% of the energy was saved thanks to the introduction of the air recirculation control strategy.

During test #7a, the cabin temperature reached the set-point temperature only at the end of the test (2400 s). In test #8a, the implementation of the air recirculation control strategy allowed the reaching of the cabin set-point temperature after 1300 s. As shown in Figure 5b, we can see some oscillations in the cabin temperature curve of test #8a due to the opening and closing of the fresh air flow. At the end of the experiment, the energy consumptions in test #7a and test #8a were, respectively, 0.818 kWh and 0.744 kWh. Therefore, the air recirculation control strategy allowed us to save 9.0% of the energy consumed.

### 3.2. Evaluation of the Optimal CO_2_ Sensor Position

#### 3.2.1. CO_2_ Stratification in the Breathing Zone

**Figure 6:**
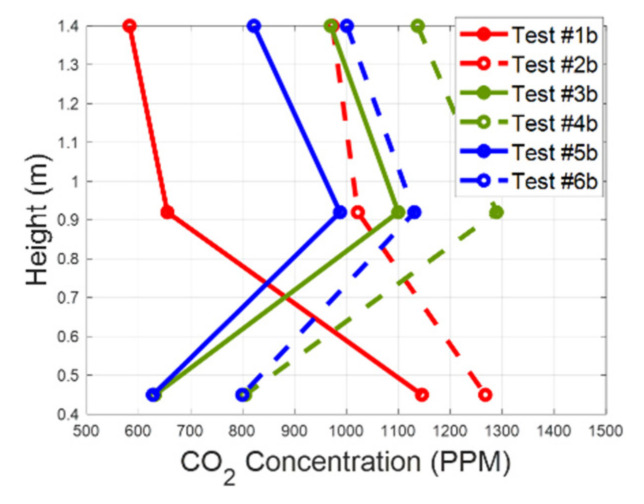
Vertical stratification of the CO_2_ in the breathing zone.

Test #1b and test #2b were executed in heating mode, and all the air was blown by the fan through the vents placed under the seat of the passenger. In contrast, in the ventilation and cooling modes the same air flow was also blown through the vents at the left of the manikin. In Figure 6 are represented the values of the CO_2_ concentration measured in the breathing zone against the height of the installation of the sensors with respect to the floor level. For the experiments where the HVAC system operated in the fresh air mode, we plotted the value of the CO_2_ concentration measured when we reached the steady-state condition for Equation (5). For the tests where the air recirculation control strategy was activated, the evolution in time of the CO_2_ concentration was always in a dynamic condition. Therefore, we plotted the time-averaged values measured by sensors 6, 7, and 8. The results in Figure 6 show different vertical stratifications of the CO_2_ levels in the breathing zone. In the heating mode, the CO_2_ concentration increased going from the roof level to the floor level, while in the ventilation and cooling modes the maximum values of the CO_2_ concentration were measured near the manikin’s shoulder level. It seems that in the last case, the air was more stationary in the breathing zone, causing an accumulation of CO_2_ in that area. Figure 6 shows that the CO_2_ stratification profile was similar in the ventilation and in the cooling modes because the air blown by the fan was distributed to the same vents. So, the internal aerodynamic of the cabin was almost alike; the difference was due to the convection effect. We can say that the diffusion of the CO_2_ in the cabin compartment is principally dependent on how we distribute the air flow between the various cabin vents.

#### 3.2.2. CO_2_ Sensor Positioning Performance

Table 5 shows the calculated values of the parameter ${\bar{\Delta }}_{i}$ which is an indicator of the difference, due to the sensor position, between the CO_2_ measurements performed by sensors 1 to 5 and the CO_2_ concentration measured in the breathing zone (sensors 6, 7, and 8). For the evaluation of the optimal sensor positioning, we considered the tests performed for the cases of internal ventilation in the fresh air mode and with the air recirculation automatically controlled, for each operating condition: the heating, ventilation, and cooling modes.

**Figure 7:**
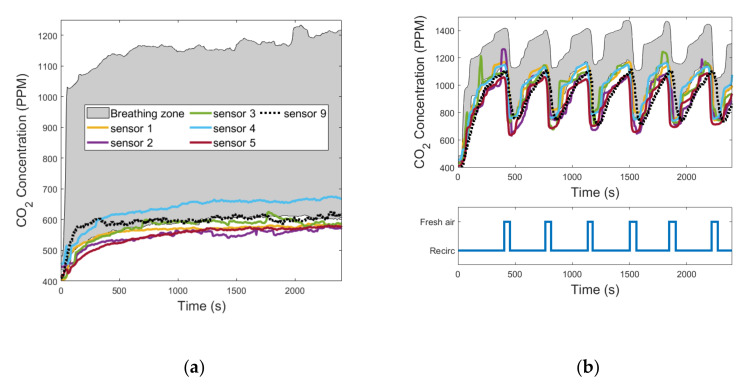
CO_2_ concentration measurements acquired from the 9 sensors that were installed in the cabin compartment: (**a**) test #1b; (**b**) test #2b (the colors of the curves are referred to in the legend in Figure 7a).

**Figure 8:**
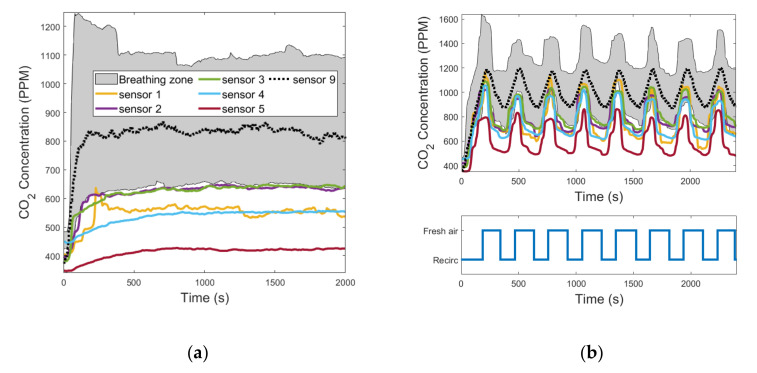
CO_2_ concentration measurements acquired from the 9 sensors that were installed in the cabin compartment: (**a**) test #3b; (**b**) test #4b (the colors of the curves are referred to in the legend in Figure 8a).

**Figure 9:**
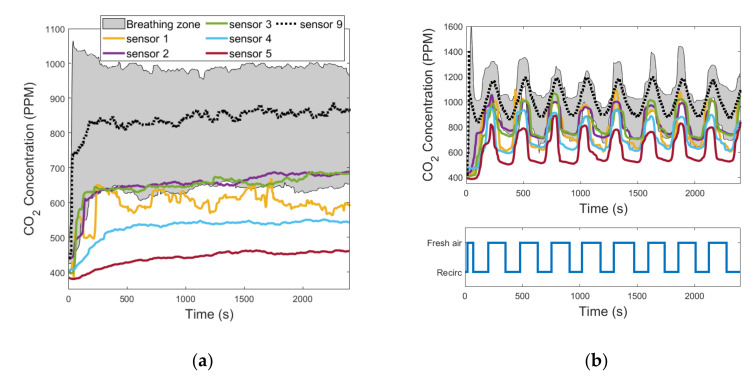
CO_2_ concentration measurements acquired from the 9 sensors that were installed in the cabin compartment: (**a**) test #5b; (**b**) test #6b (the colors of the curves are referred to in the legend in Figure 9a).

In all the tests performed, sensor 5 was the one characterized by the greatest values of ${\bar{\Delta }}_{i}$ because it was positioned in the direction of the air flow blown through the diffuser under the driver’s seat. In fact, it was always the one that recorded the lowest values of CO_2_ concentration because it was almost directly hit by the fresh air flow induced in the cabin. Figure 7a, Figure 8a, and Figure 9a show the evolution in time of the CO_2_ concentrations measured by sensors 1 to 9 in the fresh air mode. Figure 7b, Figure 8b, and Figure 9b show the same measurements performed when the automatic control of the recirculation mode was activated. When the fresh air mode was on, sensors 2 and 3, which where the nearest to the head level, were the ones that registered the lowest values of ${\bar{\Delta }}_{i}$. Instead, when the recirculation mode was on, the ones that recorded the lowest values of ${\bar{\Delta }}_{i}$ were sensors 1 and 4. Figure 7b, Figure 8b, and Figure 9b show that during test #2b, the HVAC system worked in recirculation mode for most of the time, while in test #4b and test #6b, the time spent in fresh air mode was about half the duration of the experiment. The results obtained in Table 5 were similar between the ventilation and the cooling operating conditions, while they were quite different between the heating operating condition and the other two, due to the diverse internal aerodynamic of the cabin. In the heating mode, the sensors that recorded the lowest values of ${\bar{\Delta }}_{i}$ were sensors 1 and 4, while in the other two operating conditions were sensors 2 and 3. Considering the mean between the values of ${\bar{\Delta }}_{i}$ calculated in tests #1b, #2b, #3b, #4b, #5b, and #6b, the minimum result was calculated for sensor 3, and it was 1.69%. Therefore, we concluded that the position of sensor 3 was the best one for this cabin.

### 3.3. Results of the Monte Carlo Method Applied on Indirect Measurements

**Figure 10:**
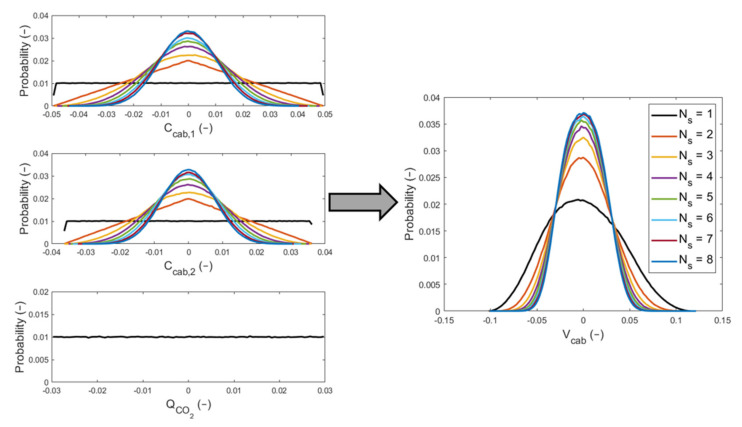
Illustration of the propagation of distribution for the input quantities obtained by the application of the MCM in test #1c.

When two CO_2_ sensors are used, the probability distribution of the variables ${\hat{C}}_{cab,1}$ and ${\hat{C}}_{cab,2}$ becomes triangular, while with three or more CO_2_ sensors, it becomes Gaussian. The results from test #1c showed that the probability distribution of ${\hat{V}}_{cab}$ is Gaussian in all the tests performed, with a decreasing standard deviation as the number of CO_2_ sensors considered ($N_{s}$) increases. In Figure 10, the propagation of distributions for the input variables that appear in Equation (7) is plotted. On the left, there are the probability distributions for the input quantities: the in-cabin CO_2_ concentration levels at $t_{1}$ and $t_{2}$ and the source of CO_2_. On the right, there is the probability distribution for the indirect measurement of test #1c, which is the cabin volume. The curves related to each value of $N_{s}$ are plotted, and the results in the x-axis of Figure 10 have been normalized in this way:

(12) $$x^{*}=\frac{x-\mu_{x}}{\mu_{x}}$$

where x is a generic variable and $\mu_{x}$ is the mean value of x.

Table 6 lists the results from test #1c performed by using the MCM. The values contained in the table are the mean value and the standard deviation for the variables ${\hat{C}}_{cab,1}$, ${\hat{C}}_{cab,2}$, ${\hat{Q}}_{CO_{2}}$, and ${\hat{V}}_{cab}$. They were found for the eight cases studied where the number of the CO_2_ sensor considered in the cabin was changed from 1 to 8. The standard deviation values have been written with one significant digit, as suggested by the GUM [22]. For $N_{s}$ greater than 4, it is not possible to observe changes in $\sigma_{V_{cab}}$.

**Figure 11:**
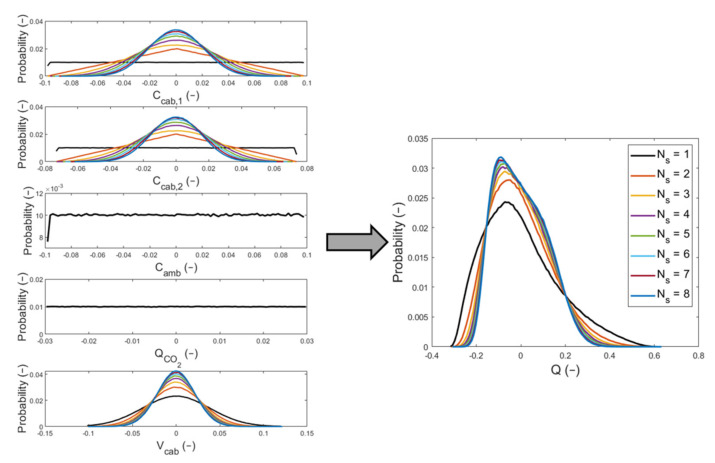
Illustration of the propagation of distribution for the input quantities obtained by the application of the MCM in test #2c.

The findings obtained from test #1c for the variable ${\hat{V}}_{cab}$ were used in test #2c to define the Gaussian distribution from which the element ${\hat{V}}_{cab}\left(m\right)$ was sampled at each Monte Carlo trial. The outcomes found from test #2c revealed that the probability distribution of $\hat{Q}$ is not symmetric around the mean value $\mu_{Q}$. In Figure 11, the propagation of distributions for the variables that appear in Equation (10) is plotted. On the left, there are the probability distributions for the input quantities: the in-cabin CO_2_ concentration levels at $t_{1}$ and $t_{2}$, the CO_2_ concentration levels in the external ambient conditions, the source of CO_2_, and the cabin volume. On the right, there is the probability distribution for the indirect measurement of test #2c, which is the leakage flow. Moreover, in this figure the curves related to each value of $N_{s}$ are plotted, and the results have been normalized as defined in Equation (12).

The results obtained from test #2c, by using the MCM, are listed in Table 7 and Table 8. Moreover, in this case the standard deviations values have been written with one significant digit. In Table 7, the mean value and the standard deviation of the input variables are recorded, for the $N_{s}$ values equal to 1 to 8: ${\hat{C}}_{cab,1}$, ${\hat{C}}_{cab,2}$, ${\hat{C}}_{amb}$, ${\hat{Q}}_{CO_{2}}$, and ${\hat{V}}_{cab}$.

As $\hat{Q}$ was not symmetrically distributed around the mean value, we used the shortest 95% coverage interval to define its measurement uncertainty. In Table 8, we listed the estimate $\mu_{Q}$ and the endpoints of the coverage interval for the leakage flow (the output variable).

Every time we increase the number of CO_2_ sensors installed into the cabin, the benefit in terms of the measurement uncertainty improvement of the leakage flow is smaller. Hence, at a certain point, it is not convenient to add other CO_2_ sensors, considering that we are increasing the cost of the HVAC system. Increasing the value of $N_{s}$, the 95% coverage interval tended to become symmetric around the mean value $\mu_{Q}$, until $N_{s}$ was equal to 7. When $N_{s}$ was equal to 8, a shift to the right of the 95% coverage interval was observed. The reported results in Table 8, corresponding to declaring one significant digit in the standard uncertainty, show that we cannot appreciate a change in the 95% coverage interval for $N_{s}$ greater than 4, apart from when $N_{s}$ was equal to 8. Even in Figure 11, we can see that the curves of the probability distributions for $N_{s}$ greater than 4 are pretty much superimposed. Therefore, we think that it is not convenient to install more than four CO_2_ sensors inside the cabin compartment.

## 4. Discussion

We all know that with the recirculating of the cabin air it is possible to decrease the HVAC system energy consumption to maintain a certain target internal condition, thanks to the smaller temperature difference between the air at the inlet and at the outlet of the HVAC unit. However, the literature shows how fast the CO_2_ concentration levels can rise in a vehicle cabin compartment when the air is recirculated, or the fan is turned off. Furthermore, if no fresh air is induced into the cabin, the CO_2_ concentration can reach levels that can have effects on the health of the driver and the passengers. Some of the effects, due to a prolonged exposition to high CO_2_ concentration levels, can be headache, drowsiness, and decrements in decision-making performance. Compared to the passenger vehicles used by other researchers, the CO_2_ measurements that we performed in our crane cabin revealed a slower increase in the CO_2_ concentration levels. This means that a crane cabin is less hermetic than a generic passenger vehicle cabin. In the present study, we implemented a control strategy for the air recirculation that allowed a reduction in the HVAC system energy consumption, while keeping the cabin CO_2_ concentration under 1100 ppm. We also paid attention to the propagation of the CO_2_ inside the cabin volume, in different operating modes, because generally it is not uniformly distributed. The results showed that the latter is mainly dependent on how the air flow coming from the HVAC unit is spread to the various cabin vents. As we were interested to maintain a safe condition regarding the CO_2_ concentration levels in the breathing zone, the ventilation setting influenced the demanded fresh air flow. Consequently, it affected the energy saving due to the execution of the air recirculation control strategy. As in a real application we cannot place the CO_2_ sensor in the breathing zone, we studied five potential spots for the sensor. The purpose was to determine in which of these positions we could measure the closest CO_2_ concentration levels to the breathing zone, in the operating modes analysed. By defining a parameter that compared the CO_2_ measurements in these five locations with the ones in the breathing zone, we found that the spot at the top right space of the cabin was the optimal one. Finally, considering that some standards related to vehicle air conditioning define the minimum fresh air flow that must be introduced in the cabin compartment, we used the MCM to compute the measurement uncertainty of the leakage flow. In particular, we observed how the confidence interval for the leakage flow changed depending on the number of CO_2_ sensors installed inside the cabin. The confidence interval depends on the accuracy of each sensor and on the number of sensors used. In this regard, we made tests varying the number of sensors considered while keeping unchanged the type of sensors (in other words the accuracy of each sensor), and we found that using more than four sensors did not produce an appreciable further improvement in the confidence interval. To sum up, we can assume four as the optimal number of CO_2_ sensors to improve the accuracy of our measurement.

In the future research, we will investigate the impact of the accuracy of the CO_2_ sensors used on the HVAC system energy consumption. Another topic of interest to investigate is whether it is possible to reduce the costs while achieving the same CO_2_ measurement accuracy, using a larger number of cheaper sensors.

## Figures and Tables

**Table 1:** Settings of the climatic wind tunnel and of the HVAC system for the tests executed in the evaluation of the energy saved thanks to the implementation of a controlled outside air induction.

Test #	T_amb_ ^1^ [°C]	W_rad_ ^2^ [W/m^2^]	RH ^3^ [%]	V_wind_ ^4^ [km/h]	Working Mode	Recirc. Flap	S_CO2_ ^5^ [g/h]	T_set-point_ ^6^ [°C]
1a	10	590	70	15	Heating	Fresh air	66	21
2a						Auto ^7^		
3a	15	590	60	15	Heating	Fresh air	66	21
4a						Auto		
5a	23	590	55	15	Cooling	Fresh air	66	21
6a						Auto		
7a	30	685	50	15	Cooling	Fresh air	66	21
8a						Auto		

^1^ Ambient temperature. ^2^ Solar radiation. ^3^ Relative humidity. ^4^ Wind speed. ^5^ CO_2_ generation. ^6^ Cabin set-point temperature. ^7^ Automatically controlled: the HVAC control unit switches the position of the flap from the recirculation mode to the fresh air mode position depending on the CO_2_ concentration level measured.

**Table 2:** Settings of the climatic wind tunnel and of the HVAC system for the tests executed in the evaluation of the optimal CO_2_ sensor positioning.

Test #	T_amb_ [°C]	W_rad_ [W/m^2^]	RH [%]	V_wind_ [km/h]	Working Mode	Recirc. Flap	S_CO2_ [g/h]	T_set-point_ [°C]
1b	15	590	60	15	Heating	Fresh air	66	21
2b						Auto ^7^		
3b	20	590	55	15	Vent. on	Fresh air	66	-
4b					Vent. on	Auto		
5b	30	685	50	15	Cooling	Fresh air	66	21
6b						Auto		

**Table 3:** Settings of the climatic wind tunnel and of the HVAC system for the analysis made with the MCM.

Test #	T_amb_ [°C]	W_rad_ [W/m^2^]	RH [%]	V_wind_ [km/h]	V_fan_ ^1^ [%]	Recirc. flap
1c	20	0	55	0	0	-
2c				15	50	Fresh air

^1^ Fan speed, expressed as % of its maximum value.

**Table 4:** Errors relative to the input quantities for test #1c and test #2c.

Test #	$e_{C_{i,1}}{\;}^{1}\;(\mathrm{ppm})$	$e_{C_{i,2}}{\;}^{1}\;(\mathrm{ppm})$	$e_{C_{amb}}{\;}^{1}\;(\mathrm{ppm})$	$e_{Q_{CO_{2}}}{\;}^{1}\;(m^{3}/s)$	$\sigma_{V_{cab}}{\;}^{2}\;(m^{3})$
1c	77	171	-	3 × 10^−7^	-
2c	43	51	43	3 × 10^−7^	Found from test #1c

^1^ $e_{j}=\left(b_{j}-a_{j}\right)/2$^2^ This value was found from Test #1c, and it is shown in Table: Results from test #1c using MCM; as shown in Section 3.3, the cabin volume PDF was Gaussian, so we indicated its standard deviation.

**Table 5:** Values of ${\bar{\Delta }}_{i}$ for CO_2_ sensors 1 to 5.

	Heating Mode		Ventilation Mode		Cooling Mode	
Sensor	Fresh Air [%]	Auto [%]	Fresh Air [%]	Auto [%]	Fresh Air [%]	Auto [%]
1	2.57	1.20	9.10	5.82	4.32	4.61
2	2.49	7.22	1.36	2.79	0.15	1.54
3	1.31	3.23	1.51	1.72	0.17	2.19
4	0.00	1.56	10.54	6.45	12.22	8.87
5	4.47	8.64	24.24	19.15	22.84	18.97

**Table 6:** Results from test #1c using MCM.

$N_{s}$	$\mu_{C_{cab,1}}\;(\mathrm{ppm})$	$\sigma_{C_{cab,1}}\;(\mathrm{ppm})$	$\mu_{C_{cab,2}}\;(\mathrm{ppm})$	$\sigma_{C_{cab,2}}\;(\mathrm{ppm})$	$\mu_{Q_{CO_{2}}}\;(m^{3}/s)$	$\sigma_{Q_{CO_{2}}}\;(m^{3}/s)$	$\mu_{V_{cab}}\;(m^{3})$	$\sigma_{V_{cab}}\;(m^{3})$
1	1560	40	4700	100	1.00 × 10^−5^	2 × 10^−7^	2.23	0.09
2	1560	30	4700	70	1.00 × 10^−5^	2 × 10^−7^	2.23	0.07
3	1560	30	4700	60	1.00 × 10^−5^	2 × 10^−7^	2.23	0.06
4	1560	20	4700	50	1.00 × 10^−5^	2 × 10^−7^	2.23	0.05
5	1560	20	4700	40	1.00 × 10^−5^	2 × 10^−7^	2.23	0.05
6	1560	20	4700	40	1.00 × 10^−5^	2 × 10^−7^	2.23	0.05
7	1560	20	4700	40	1.00 × 10^−5^	2 × 10^−7^	2.23	0.05
8	1560	20	4700	30	1.00 × 10^−5^	2 × 10^−7^	2.23	0.05

**Table 7:** Results for the input variables from test #2c, using MCM.

$N_{s}$	$\mu_{C_{cab,1}}\;(\mathrm{ppm})$	$\sigma_{C_{cab,1}}\;(\mathrm{ppm})$	$\mu_{C_{cab,2}}\;(\mathrm{ppm})$	$\sigma_{C_{cab,2}}\;(\mathrm{ppm})$	$\mu_{C_{amb}}\;(\mathrm{ppm})$	$\sigma_{C_{amb}}\;(\mathrm{ppm})$	$\mu_{Q_{CO_{2}}}\;(m^{3}/s)$	$\sigma_{Q_{CO_{2}}}\;(m^{3}/s)$	$\mu_{V_{cab}}\;(m^{3})$	$\sigma_{V_{cab}}\;(m^{3})$
1	440	20	690	30	440	20	1.00 × 10^−5^	2 × 10^−7^	2.23	0.09
2	440	20	690	20	440	20	1.00 × 10^−5^	2 × 10^−7^	2.23	0.07
3	440	10	690	20	440	20	1.00 × 10^−5^	2 × 10^−7^	2.23	0.06
4	440	10	690	10	440	20	1.00 × 10^−5^	2 × 10^−7^	2.23	0.05
5	440	10	690	10	440	20	1.00 × 10^−5^	2 × 10^−7^	2.23	0.05
6	440	10	690	10	440	20	1.00 × 10^−5^	2 × 10^−7^	2.23	0.05
7	438	9	690	10	440	20	1.00 × 10^−5^	2 × 10^−7^	2.23	0.05
8	438	9	690	10	440	20	1.00 × 10^−5^	2 × 10^−7^	2.23	0.05

**Table 8:** Results for the output variable from test #2c, using MCM.

$N_{s}$	$\mu_{Q}\;(m^{3}/h)$	Coverage Interval	
		Lower Endpoint (m^3^/h)	Upper Endpoint (m^3^/h)
1	140	110	190
2	140	110	180
3	140	110	180
4	140	110	170
5	140	110	170
6	140	110	170
7	140	110	170
8	140	120	170

## Data Availability

Not applicable.
